# Evaluation of novel precision viticulture tool for canopy biomass estimation and missing plant detection based on 2.5D and 3D approaches using RGB images acquired by UAV platform

**DOI:** 10.1186/s13007-020-00632-2

**Published:** 2020-07-03

**Authors:** Salvatore Filippo Di Gennaro, Alessandro Matese

**Affiliations:** Institute of BioEconomy, National Research Council (CNR-IBE), Via G. Caproni, 8, 50145 Florence, Italy

**Keywords:** Unmanned aerial vehicle, Precision viticulture, 3D model, Missing plants, Canopy biomass

## Abstract

**Background:**

The knowledge of vine vegetative status within a vineyard plays a key role in canopy management in order to achieve a correct vine balance and reach the final desired yield/quality. Detailed information about canopy architecture and missing plants distribution provides useful support for farmers/winegrowers to optimize canopy management practices and the replanting process, respectively. In the last decade, there has been a progressive diffusion of UAV (Unmanned Aerial Vehicles) technologies for Precision Viticulture purposes, as fast and accurate methodologies for spatial variability of geometric plant parameters. The aim of this study was to implement an unsupervised and integrated procedure of biomass estimation and missing plants detection, using both the 2.5D-surface and 3D-alphashape methods.

**Results:**

Both methods showed good overall accuracy respect to ground truth biomass measurements with high values of R^2^ (0.71 and 0.80 for 2.5D and 3D, respectively). The 2.5D method led to an overestimation since it is derived by considering the vine as rectangular cuboid form. On the contrary, the 3D method provided more accurate results as a consequence of the alphashape algorithm, which is capable to detect each single shoot and holes within the canopy. Regarding the missing plants detection, the 3D approach confirmed better performance in cases of hidden conditions by shoots of adjacent plants or sparse canopy with some empty spaces along the row, where the 2.5D method based on the length of section of the row with lower thickness than the threshold used (0.10 m), tended to return false negatives and false positives, respectively.

**Conclusions:**

This paper describes a rapid and objective tool for the farmer to promptly identify canopy management strategies and drive replanting decisions. The 3D approach provided results closer to real canopy volume and higher performance in missing plant detection. However, the dense cloud based analysis required more processing time. In a future perspective, given the continuous technological evolution in terms of computing performance, the overcoming of the current limit represented by the pre- and post-processing phases of the large image dataset should mainstream this methodology.

## Introduction

Vineyards are highly heterogeneous due to structural factors mediated by topography and soil characteristics, and non-structural factors, mediated by crop practices. Remote sensing technologies have been successfully used for vineyard monitoring and could be useful to describe vineyard variability. Unmanned Aerial Vehicles (UAV) provide high flexibility of use, low operational costs and very high spatial resolution Matese et al. [17]. RGB sensors mounted on UAVs are capable of providing high-resolution images that can be processed to build digital surface models (DSMs), using three-dimensional (3D) reconstruction software based on stereo vision or structure from motion (SfM) algorithms Padua et al. [16, 22]. Using these methods, a large set of applications can be undertaken such as biomass monitoring [4–6], volume characterization Ballesteros et al. [3], Matese et al. [15], Pádua et al. [21] and early-season crop monitoring [10], [26]. Many authors reported that the use of SfM from UAV-images may produce a 3D point cloud similar to one obtained acquiring data with a LiDAR [12, 29]. Photogrammetric dense point cloud has a point density depending on the image spatial resolution and overlap level, but with a consistently lower cost than a LiDAR one. These advantages have led to an increasing interest in this technology and in the last few years, several studies utilized dense point clouds from SfM in vineyards with different applications Ballesteros et al. [3, 14, 30].

Mesas-Carrascosa et al. [19] applied colour vegetation indices in point clouds for the automatic detection and classification of points representing vegetation and calculated the height of vines using as a reference the heights of points classified as soil.

Anifantis et al. [2] performed a comparison on an adult super-high-density olive orchard, using three methods for tree row volume (TRV). The first method (TRV1) was based on close-range photogrammetry from UAVs, the second (TRV2) was based on manual in situ measurements, and the third (TRV3) was based on a formula from the literature.

Comba et al. [9] proposed an innovative unsupervised algorithm for vineyard detection and vine row features evaluation, based on 3D point-cloud maps processing. The main results are automatic detection of the vineyards and local evaluation of vine row orientation and inter-row spacing. The overall point-cloud processing algorithm can be divided into three mains steps: (1) precise local terrain surface and height evaluation of each point of the cloud, (2) point-cloud scouting and scoring procedure on the basis of a new vineyard likelihood measure and lastly, (3) detection of vineyard areas and local features evaluation.

Comba et al. [8] used a data fusion approach to achieve high consistency of the obtained huge data for vigour characterization in vineyards using 2.5D multispectral aerial imagery, 3D point cloud crop models and aerial thermal imagery.

Missing plants in a vineyard is a critical issue that can be managed by new technologies. Different events such as disease, winter injury or mechanical damage cause missing plants over the years and the initial number of vines per hectare decrease. As a consequence, farmers lose a significant percentage of potential vineyard production. The simplest approach to identifying missing plants would be to detect areas not covered by canopy along the row. Unfortunately, vertical aerial photography is often unable to identify the actual situation under the top of the canopy, and in the case of absence of a plant, neighbouring plants can extend their shoots and foliage to occupy the adjacent free space. Using a raster surface approach, the estimation of height and area from UAV measurements does not denote tangible errors because the ground-based measurements have been derived by applying the conventional geometric equation that considers trees as ellipsoid forms, which can produce inexact ground estimations. Torres-Sánchez et al. [27], reported that 3-D products derived in their study reconstructed the irregular shape of the crown, which hypothetically allows better estimation of tree volume than those derived from ground measurements. A step forward could be taken using a methodology typical for forestry applications [28], where the 3D dense cloud is reconstructed as an object shape from a set of unorganized points. Using these methods, not only the tree crown shape was estimated, but also the entire canopy.

The aim of this study was to implement an unsupervised and integrated procedure of biomass estimation and missing plants detection, using both the 2.5D-surface and 3D-alphashape methods.

Results are presented in Sect. “Results” and discussed in Sect. “Discussion”. The most significant conclusions are shown in Sect. “Methods”. The last section presents the study area and the methods used both for data acquisition and processing.

## Results

### Model validation

**Fig. 1:**
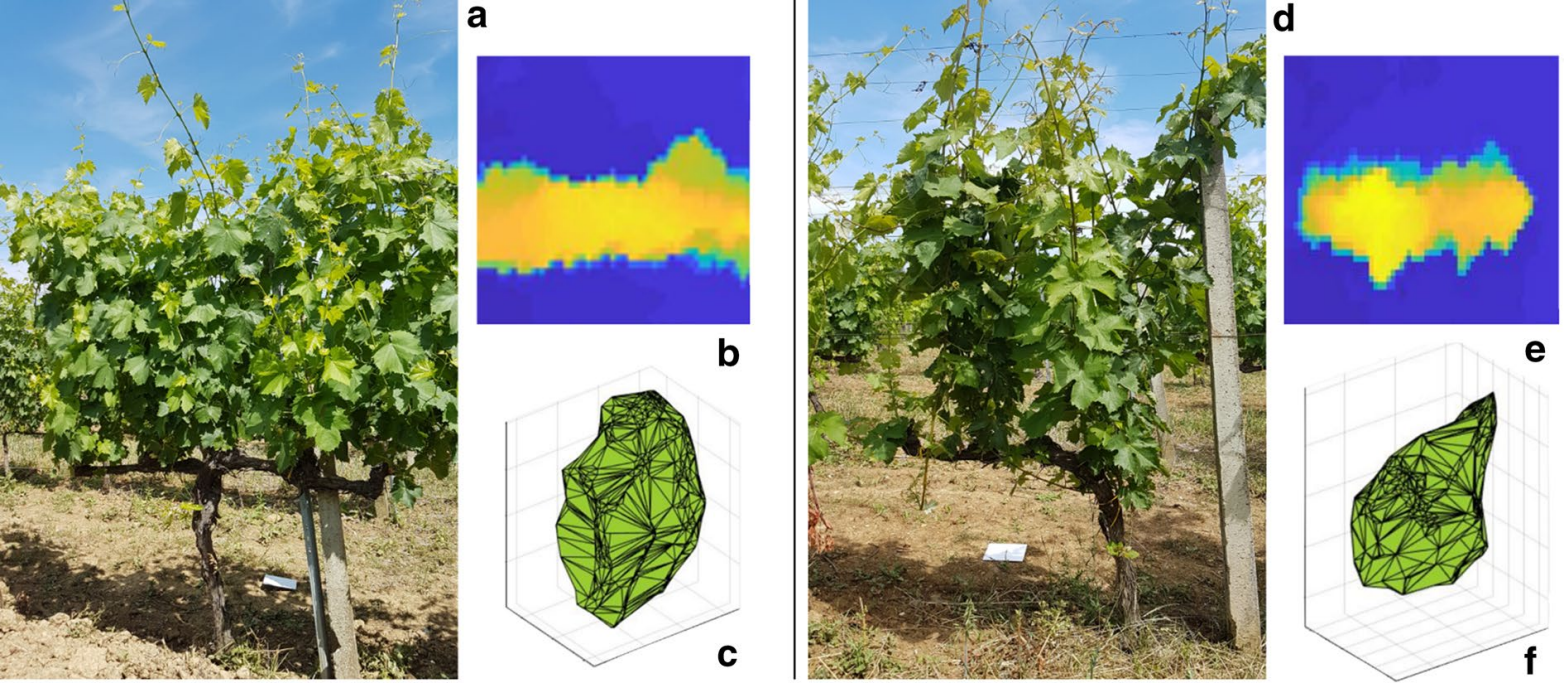
Data processing of sampled vines (**a**, **d**) using 2.5D (**b**, **e**) and 3D (**c**, **f**) methods

The model validation was performed by comparing research outputs with independent in-field observations. This phase evaluates the quantitative and qualitative accuracy and allows the comparison of alternative research methodologies. The ground-measured volume per vine was calculated using field data aiming to validate UAV 2.5D and 3D methods (Fig. 1).

**Fig. 2:**
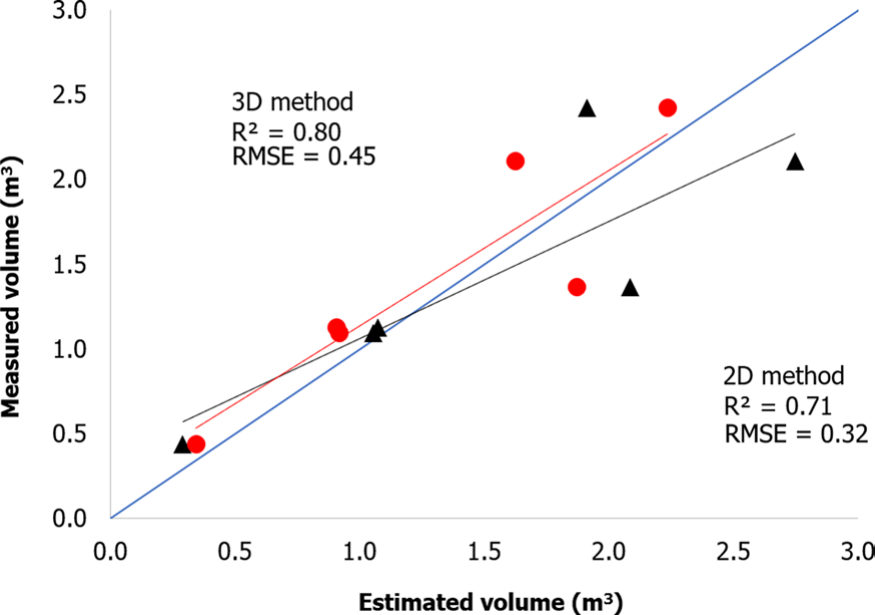
Linear regression results between vine canopy volume estimation made by 2.5D (black triangle) and 3D (red circle) methods with observed volume

Vine height and thickness are among the most used agronomic parameters by farmers for in-field volume measurements, being non-destructive and easy to acquire. These variables were therefore chosen to validate the model. Figure 2 shows the linear regression results between canopy volume estimation made by 2.5D (black triangle) and 3D (red circle) methods and volume ground measurements.

Both methods present a high value of R^2^ (0.71 and 0.80 for 2.5D and 3D, respectively) confirming the good accuracy of the models, with values distribution very close to the 1:1 line. In some cases, 2.5D method tends to overestimate since it is derived by applying the conventional geometric equation that considers the vine as rectangular cuboid form. On the contrary, the 3D method tends to underestimate canopy volume as a consequence of the alphashape algorithm, which produces shapes more complex than a rectangular cuboid taking in account the irregular shapes and detecting each single shoot and holes within the canopy.

### Biomass estimation and missing plants detection

**Fig. 3:**
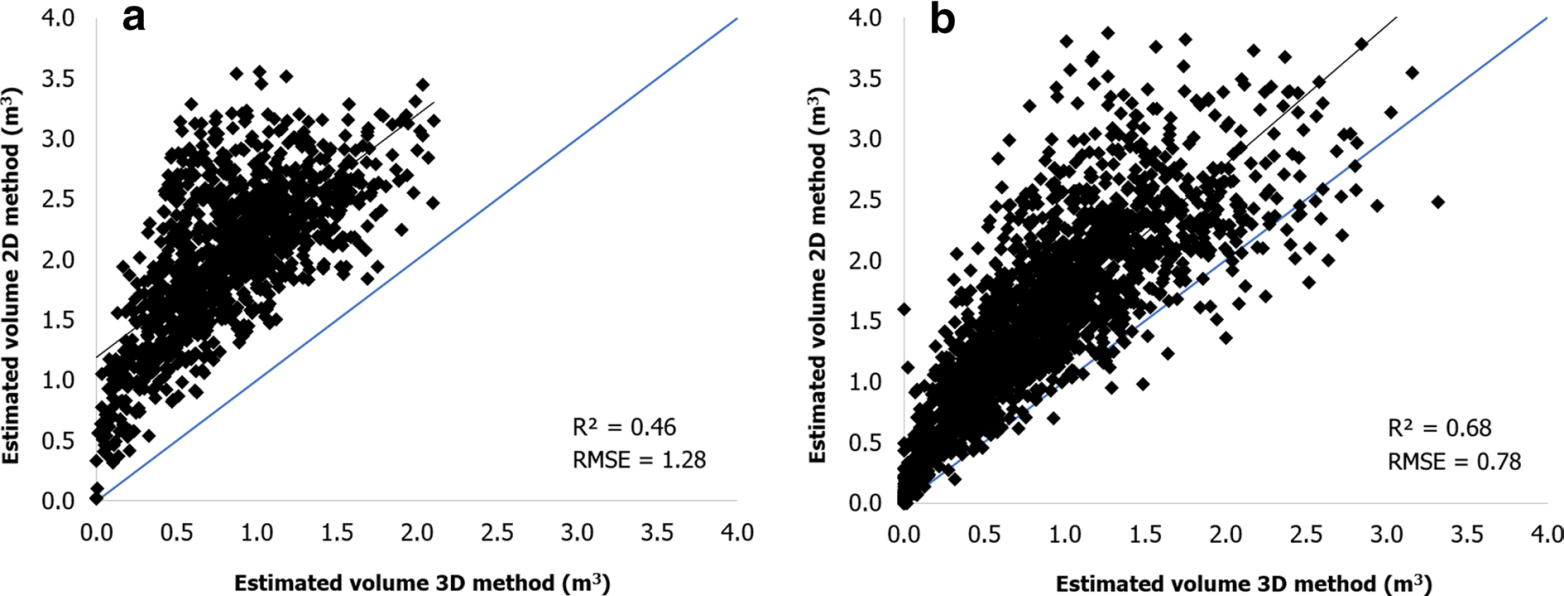
Linear regression between volume estimation of each polygon grid obtained with 3D and 2.5D methods for both sites **a** Fontone and **b** Case Basse

The vines volume within each polygon grid (3 plants) of the whole vineyard was calculated using the 2.5D and 3D method. As a consequence of the high heterogeneity of this vineyard in terms of plant age, vine spacing and canopy management, the analysis was performed separating the northern (Fontone) from the southern site (Case Basse). First, a comparison between the two methods of canopy volume estimation is shown in Fig. 3. The values represent the canopy volume estimation of all polygons grid in the two sites. In line with the results of the model validation section, both sites show a biomass overestimation of the 2.5D with respect to the 3D method, less marked in Case Basse than Fontone, being closer to the 1:1 line.

The comparison between the two methods provided an overall good correlation, with higher correlation coefficient and accuracy in term of values in Case Basse (R^2^ = 0.68, RMSE = 0.78 m^3^) with respect to Fontone (R^2^ = 0.46, RMSE = 1.28 m^3^). In detail, in Fontone (Fig. 3a) the regular geometry of the rows and the larger dimension of the canopy top derived from the shoot wrapping management cause a higher estimation of the mean canopy volume per polygon grid in the 2.5D method (2.03 ± 0.63 m^3^), which takes into account the improved canopy thickness. In this case the 3D approach presents lower mean values (0.83 ± 0.43 m^3^), evaluating the presence of holes and canopy thickness reduction in the middle part of the canopy. Case Basse site, which has a diffuse heterogeneity due first of all to the absence of canopy management, presents a scattered distribution and higher volume values per polygon grid (Fig. 3b). As for Fontone, in this site the 2.5D method provides higher mean values (1.46 ± 0.84 m^3^) than the 3D approach (0.84 ± 0.60 m^3^). The 3D method shows similar mean values of canopy volume in both sites, with a higher standard deviation error in the Case Basse site, in line with the characteristics described previously.

Table 1 summarizes the results obtained with the two methodologies within each site. The first column reports the potential number of plants calculated on the basis of vine spacing (Fontone 4444 plant/ha, Case Basse 3333 plant/ha) and site surface values. The next three columns show the cumulative amount of missing plants estimated by the 2.5D and 3D methods, and monitored on the ground, followed by the percentage accuracy obtained by the two methods with respect to ground truth observations. Finally, the last two columns present the mean canopy volume (m^3^/vine) estimated by the two methods, calculated from the total canopy volume derived from the sum of each row volume, divided by the number of plants detected (potential plants minus estimated missing plants).

**Table 1:** Results of missing plants (MP) and canopy volume per vine (CVol) estimation for each site using 2.5D and 3D methods

Site	Potential plants	2.5D estimated MP	3D estimated MP	Ground observed MP	2.5D accuracy (%)	3D accuracy (%)	2.5D estimated CVol (m^3^/vine)	3D estimated CVol (m^3^/vine)
Fontone	3111	446	553	521	−14.40	+ 6.14	0.79	0.34
Case basse	6233	2247	2171	1931	+ 16.36	+ 11.05	0.72	0.41

Fontone shows a total of 521 missing plants counted with ground observations, the 2.5D method underestimates with a total of 446 missing plants (−14.40%), while the 3D method overestimates observed value with a total of 553 missing plants detected (+6.14%). In the Case Basse site with a total of 1931 missing plants monitored on the ground, the 2.5D and 3D methods provide an overestimation of 16.36% and 11.05%, respectively.

Fontone site has significantly fewer missing plants than Case Basse, mainly due to the smaller surface area (0.7 ha Fontone and 1.9 ha Case Basse). However, considerable influence derives from the age of the vines, which in absolute values led to a percentage of missing plants monitored on the ground with respect to the potential plants of 16.75% in Fontone and 30.98% in Case Basse, planted in 1999 and 1973, respectively.

Taking into consideration the canopy volume estimation, Table 2 shows that in Fontone site the two 2.5D and 3D methods identified a mean canopy volume value of 0.79 m^3^ and 0.34 m^3^, respectively. In line with the other site, in Case Basse the 2.5D estimates higher values than 3D method, with 0.72 m^3^ compared to 0.41 m^3^.

**Table 2:** Experimental vineyard description

Site	Fontone	Case Basse
Vineyard surface	0.7 ha	1.9 ha
Row orientation	NE/SW	NE/SW
Year	1999	1973
Rows	19	36
Variety	Sangiovese	Sangiovese
Rootstock	110R	Kober 5BB
Vine training system	Cordon spur-pruned	Cordon spur-pruned
Vine spacing	2.5 x 0.9	3.0 x 1.0
Canopy management	Shoots wrapped along the row	Free shoots

## Discussion

### Biomass estimation and missing plants detection

**Fig. 4:**
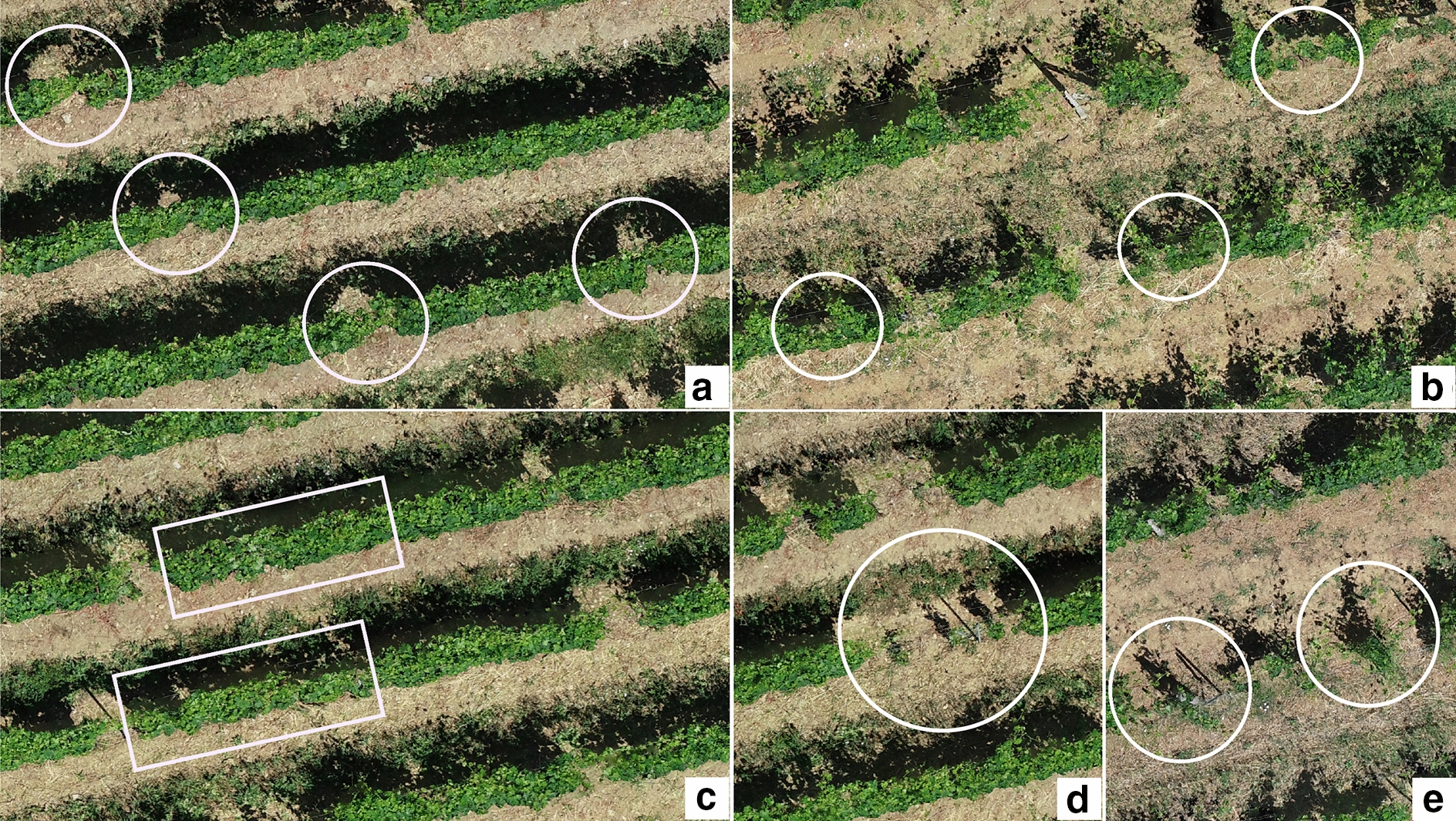
Details of vineyard conditions affecting missing plants detection in Fontone and Case Basse sites: (**a**-Fontone) false negative and (**b**-Case Basse) false positive with 2.5D method, (**c**-Fontone) false positive with 3D method due to different canopy thickness within the field and false positive with both method due to new replacement vines (**d**-Fontone and **e**-Case Basse)

The 2.5D method showed different behaviour for missing plants estimation in the two sites. In Fontone the method identified an underestimation due to the palissage technique, which caused frequent cases of missing plants hidden by adjacent plant’s shoots wrapped on the top wire of the row. In the case of a missing plant covered by vigorous shoots, the 2.5D method based on the length of section of the row with lower thickness than the threshold used (0.10 m), tended to return false negatives (Fig. 4a). On the contrary, in Case Basse site the 2.5D approach overestimated the number of missing plants with many false positives, because different spacing between vines causes numerous short non-vegetated sections, while the dispersed canopy with many lateral shoots leads to low vegetated canopy along the rows, which are frequently below the thickness threshold of the method (Fig. 4b). Instead, the 3D method, identifying missing plants on the basis of mean average canopy volume, managed to recognize the entire canopy volume including lateral shoots, and therefore provided fewer false positives than 2.5D method in the case of short interruptions or sections of reduced thickness. The irregular conditions of Case Basse led to a higher error in missing plants estimation than in Fontone site, where the palissage cover didn’t affect the accuracy of the 3D methodology, which correctly identifies the empty space of a missing plant under the wrapped shoots of adjacent plants. However, the 3D method showed a general overestimation, which derived from the need to set an average volume value of the canopy in the phase preliminary to data processing. So, in the case of areas with low vigour plants, where the canopy volume is much lower than the set threshold value, the method could return false positives. In fact, if the total volume of 3 plants in a polygon grid was lower than the volume of two average plants, it would identify the presence of a missing plant (Fig. 4c). This could be considered as the main limitation of the 3D methodology in high spatial variability conditions, however, this issue could be overcome by finding representative polygon grids without missing plants and calculating the average vine volume value. Some new replacement vines within both sites provide an additional increase of false positives cases in both sites (Fig. 4d Fontone and Fig. 4e Case Basse).

The different canopy architectures in the two study sites caused a volume overestimation applying the 2.5D method due to the increasing estimation of vine thickness within each polygon grid. In fact, in the Fontone site the palissage management caused greater top canopy area as a consequence of the huge number of leaves on main and lateral shoots, while in Case Basse site the free shoots extending in the inter-row zone increased the mean thickness value identified. The 3D method overcame that limitation, identifying a volume closer to the real canopy geometry in terms of lower thickness under the canopy top in Fontone, and considering the free shoots in the inter-row for their real volume in Case Basse, while in the 2.5D method there was a high increase in the rectangular cuboid formula. Unfortunately, to validate the average canopy volume estimation identified at whole field level, numerous samples of pruned wood within each site would be necessary, given the geometric differences in the two areas of the vineyard.

### General discussion

**Fig. 5:**
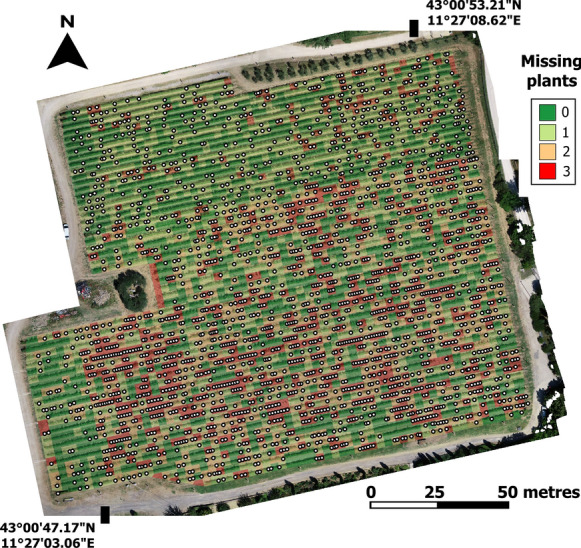
Example of graphical output of the 3D method representing a thematic map of the missing plants detected within each polygon grid, with overlaid white dots representing ground truth observations

The thorough analysis performed on the huge dataset (around 1000 vines) by the extraction of geometric parameters based on polygon grids provides a feasible and fast tool for missing plant and biomass evaluation on large areas. Furthermore, applying a surface 3D reconstruction approach, the UAV estimation of canopy height, area and volume is an objective tool with respect to subjective ground-based measurements derived by applying the conventional geometric equation that considers the trees as ellipsoid or cuboid forms, which can produce inexact ground estimations. Figure 5 shows a graphical output of the 3D method, which represents a thematic map of the missing plants detected within each polygon grid at whole vineyard level, while overlaid white dots are the ground truth observations. It could be a valuable support map for the farmer when replacing missing plants.

Previous studies have made an effort to calculate geometric variables of vines using UAV point clouds. Mesas-Carrascosa et al. [19] reported good results comparing ground measurements of the heights of individual grapevines with the estimated heights from the UAV point cloud, showing high determination coefficients (R^2^ > 0.87) and low root-mean-square error (0.07 m). For volume characterization, Anifantis et al. [2] described the calculation of tree row volume (TRV) comparing three different methodologies that show an average value of the difference equal to +13% between the method based on UAV and manual in situ measurements. Caruso et al., 2017 found a good correlation between measured and UAV estimated canopy volume, with a constant height of 0.9 m R^2^ was equal to 0.62, while R^2^ increased (0.75) when the actual distance of the canopy from the ground was used in the calculation, but in this case the estimated canopy volume diverged from the 1:1 line.

Regarding missing plant detection, Comba et al. [9] developed maps with the spatial location of classification inaccuracies in terms of over, under, extra and miss-detected areas. The good detection index was found to be always greater than 90.0%, with an overall average value on the four point-cloud maps of 94.02. Unfortunately, the validation dataset was developed manually using the high-resolution images without a robust ground truth measurement. Comba et al. [8] reported an index similar to that used in this paper, the index I3D related to canopy vigour, defined as triangulated mesh by an alphashape function, with α radius parameter equal to 0.5, calculated from the UAV 3D point cloud. The results are very interesting, showing a good classification of vines in different vigour classes using multi source data that give an improvement ranging from 67% to 90% with respect to a single data source. However, the validation dataset lacked ground measurements made by expert agronomists who classified the vineyard blocks into three classes on the basis of vigour and canopy density. De Castro et al. [10] used DSM-OBIA model for the detection of the area and height of vines, and the existence of gaps. The correct classification percentage (true positive) for each field and growth stage analyses surprisingly reached 100%. False negatives that indicated gaps wrongly classified as vines only occurred in one field, with 3.2%. Weiss and Baret [30] sampled 20 sites called elementary sampling units (ESU) covering a 10 m square area. The percentage of missing segments of rows for each ESU was computed and results showed that when the percentage of missing row segments and percentage of missing pixels are low (ESUs 1 to 4, 13, 16, 17), a very good consistency between the two methods and ground measurements is observed. [20] found high accuracy in grapevine detection (94.40%) and low error in grapevine volume estimation, and in a new work [21] this accuracy is higher (99.8%), as well as in the individual grapevine identification (mean overall accuracy of 97.5%), both works using the same method.

Puletti et al. [24] used the Red channel for identification of grapevine rows achieving acceptable accuracy values (lower than 87%), however the inter-row spaces were not vegetation-covered. In accordance with Castro et al. [10], the use of DSM and a 3D model in vine classification is shown to be more accurate than a spectral approach, especially in the challenging spectral similarity scenario due to cover crops growing in the inter-rows. The methodology presented in this study for geometric characteristic evaluation and vine classification was fully automatic compared to others that needed a manual adjustment in filtering non-vine features [13], manual detection [9] or prior training of the classifier Poblete-Echeverria et al. [23]. Although some of these approaches have a high accuracy level, they required user intervention and absence of inter-row grass cover.

However, considering the low flying quote and forward speed needed for the methodologies suggested to obtain the necessary accuracy dense clouds, the UAV battery autonomy is the main limit in terms of surface covered in a single survey. Nevertheless, the recent advances in UAV technologies provide new commercial products, which is a cost-effective solution that can cover a 3 ha vineyard in a single survey using the acquisition protocol tested in this study. Moreover, with respect to traditional spectral monitoring, a strong point of the RGB geometric analysis is the relative independence from light conditions. Consequently, the methodology suggested represents a feasible tool to monitor large areas exploiting a wide time window during the day. The main issue remains the large amount of data acquired to be processed, because image processing requires computers to be equipped with a larger working memory to reconstruct dense clouds and perform image analysis, especially following 3D methodology.

## Conclusion

This study confirmed the feasibility of a rapid assessment of biomass volume using different approaches based on the SfM algorithm applied to high resolution RGB images with large overlap acquired by a UAV platform. A secondary task was an accurate identification of missing plants within the rows, also able to detect a single dead vine, where only the cordon is present, partially covered by adjacent ones. These methods provided thematic maps related to biomass and missing plants with the aim of supporting the farmer in canopy management in order to achieve the desired vine balance. Another potential application could be optimization of the re-planting process, better quantifying the order of new vines from a nursery and allowing fast localization of each re-planting site. This paper describes a rapid and objective tool for the farmer to promptly identify canopy management strategies and drive replanting decisions. In the future, given the continuous technological evolution in terms of computing performance, this methodology could find wide diffusion eliminating the current limit represented by the pre- and post-processing phases of the large dataset of images necessary for this type of approach. Furthermore, it will also be possible to use flights with an angled camera to acquire a double dataset relating to each side of the row. This allows an extremely accurate point cloud to be obtained, but currently it is not feasible on large surfaces due both to the additional surveys needed for each vineyard, and the processing times that would be very lengthy or even impossible in the case of intermediate level workstations, given the high memory requirement for the management of such large datasets.

## Methods

### Experimental site

The research was undertaken in 2019 during fruit-set phenological stage, in a non-irrigated 2.6 ha vineyard (43.00°N, 11.26°E) located in Montalcino Domain (Siena, central Italy) on the Agricola Case Basse farm. The vineyard is divided in two parts, the south side planted in 1973 (Case Basse) and the north planted in 1999 (Fontone) (Table 2).

**Fig. 6:**
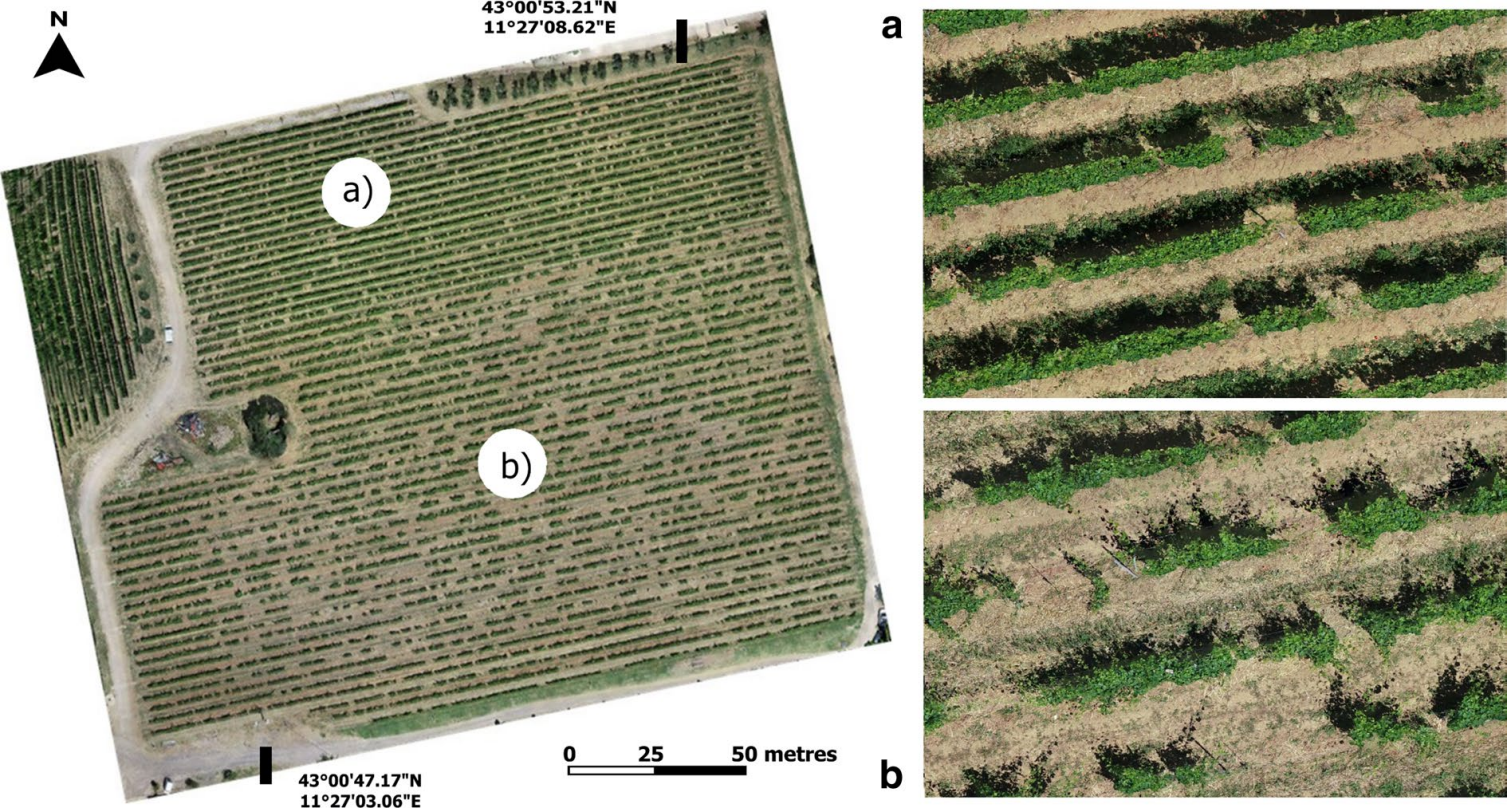
Experimental vineyard and detailed conditions of Fontone (**a**) and Case Basse (**b**) areas

This vineyard was chosen to evaluate the methodological approach under extreme and different conditions. In fact, Case Basse presents an irregular structure with different plant ages and spacing along the rows due to large number of replacements over the years, with several cases of bilateral cordon trained to cover an adjacent missing plant. On the contrary, Fontone has a regular spacing, fewer missing vines and less presence of new replacement plants (Fig. 6).

The canopy management approach used by Agricola Case Basse farm is based on the “palissage” technique, which is an alternative tool respect to the widely used hedging practice to control vine vigour. According to this canopy management, the long shoot tips that would normally be hedged are wrapped horizontally along the last catch wire on the top of the canopy. As reported by France et al. [7], this approach slowed shoot growth earlier during the season and reduced or eliminated the need for leaf removal in the fruiting zone, due to fewer lateral shoots. Other benefits are the reduction of botrytis incidence and severity because there is a better air flow through the cluster, and improved protection from hail. At flight time, the palissage technique was used only in Fontone, while in Case Basse site the long shoots were still extended in the inter-row.

### Remote sensing platform and data pre-processing

**Fig. 7:**
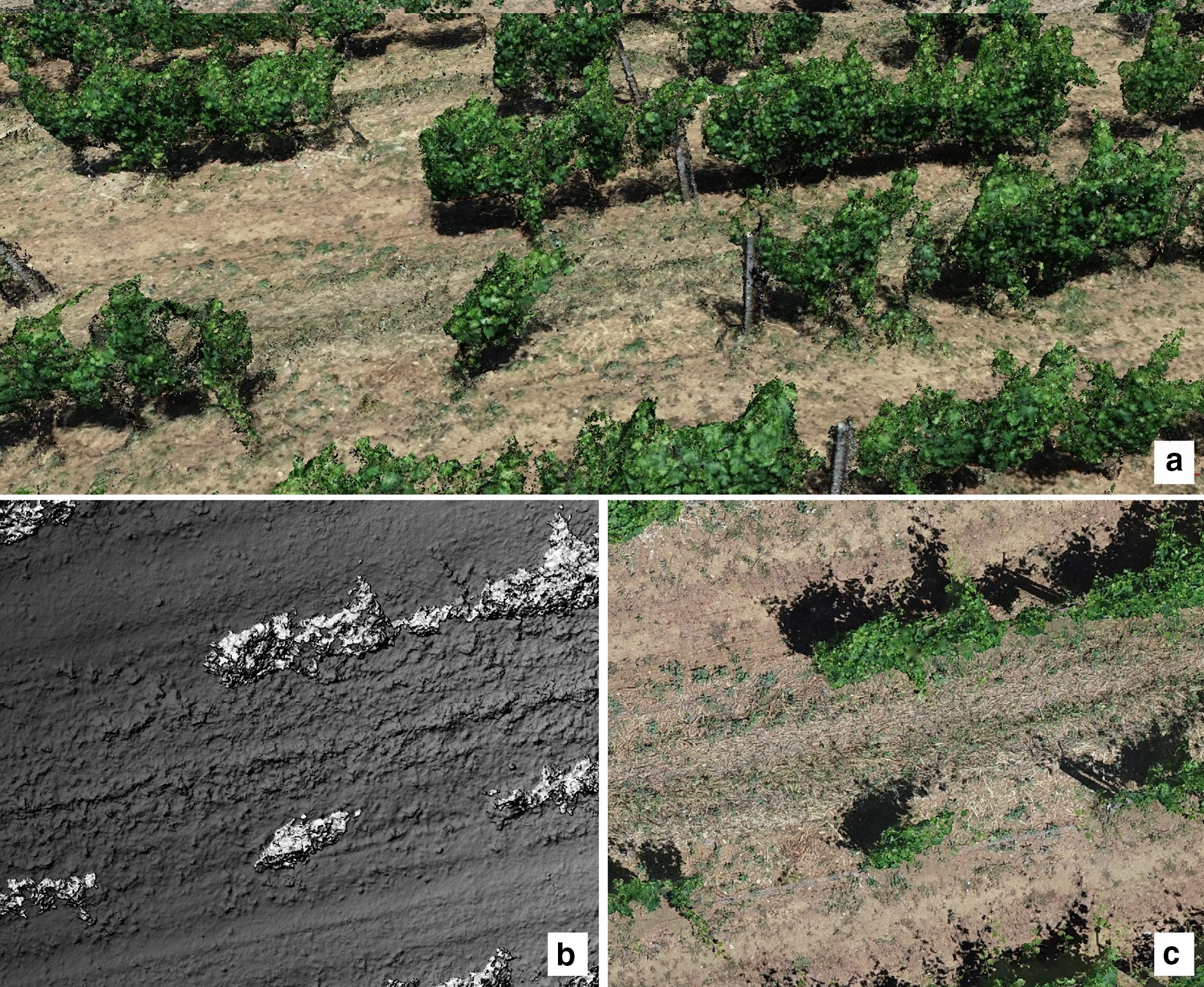
Detail of pre-processing products: dense point cloud (**a**), digital elevation model (**b**) and orthomosaic (**c**)

The flight campaign was performed using an open-source UAV multi-rotor platform consisting of a modified multi-rotor Mikrokopter (HiSystems GmbH, Moomerland, Germany) described in a previous work of the authors [16]. A universal camera mount equipped with three servomotors allowed accurate image acquisition through compensation of tilt and rolling effects. The RGB camera was a Sony Cyber-shot DSC-QX100 RGB camera (Sony Corporation, Tokyo, Japan) with a 20.2 megapixel CMOS Exmor R sensor and a Carl Zeiss Vario-Sonnar T lens. The flight campaign was performed on 20 June 2019 with a single flight survey conducted at 25 m above ground level at midday, yielding a ground resolution of 0.005 m/pixel. The RGB camera was set at 2 s automatic trigger frequency with automatic exposure. The waypoint route was generated to obtain more than 75% overlap between photos (forward overlap) and flight lines (lateral overlap), in order to achieve the highest accuracy in mosaicking elaboration step. A dataset of 501 images was used to generate pre-processed products using Agisoft Metashape Professional v.1.6.0 (Agisoft LLC, St. Petersburg, Russia). The alignment and dense point cloud elaboration steps were realized with “highest accuracy” and “ultra high quality” respectively, requiring around 10 h of computing time with a workstation equipped with two Intel Xeon E5-2690 v4 processors, 256 GB RAM and GPU Nvidia Quadro M6000 24 GB. The accurate dense point cloud (1345 million points) (Fig. 7a) was then processed to generate the digital elevation model (DEM) (Fig. 7b) and the orthomosaic (Fig. 7c) taking 40 min computing time. Data analysis computational time was 1 and 3 h for 2.5D and 3D respectively. The on-site evaluation for biomass volume was limited to sampling vines used for validation, while the missing plants ground geolocating required about 6 h.

### Ground measurements

Ground-truth measurements were performed for model validation. For vine volume assessment, they consisted of measuring canopy geometric features of 6 vigorous sample vines. In detail, canopy mean height above the cordon (CH), canopy mean length along the row (CL) and mean thickness (CT) recorded at cordon level, 0.8 m above the cordon and top of the canopy were measured. As a consequence of the heterogeneity of Case Basse site, two vines with bilateral cordon were chosen as sample vines.

The canopy volume of each vine was calculated using the following equation:

$$\text{Canopy\,volume}\, = \,\text{CH}\,{ \times }\,\text{CT}\,{ \times }\,\text{CL.}$$

The missing plant validation was performed collecting field data by visually counting and georeferencing each missing plant with a 0.02 m accuracy D-GPS (Differential-GPS LeicaGS09, Leica Geosystems, Heerbrugg, Switzerland).

### 2.5D approach–surface model method

**Fig. 8:**
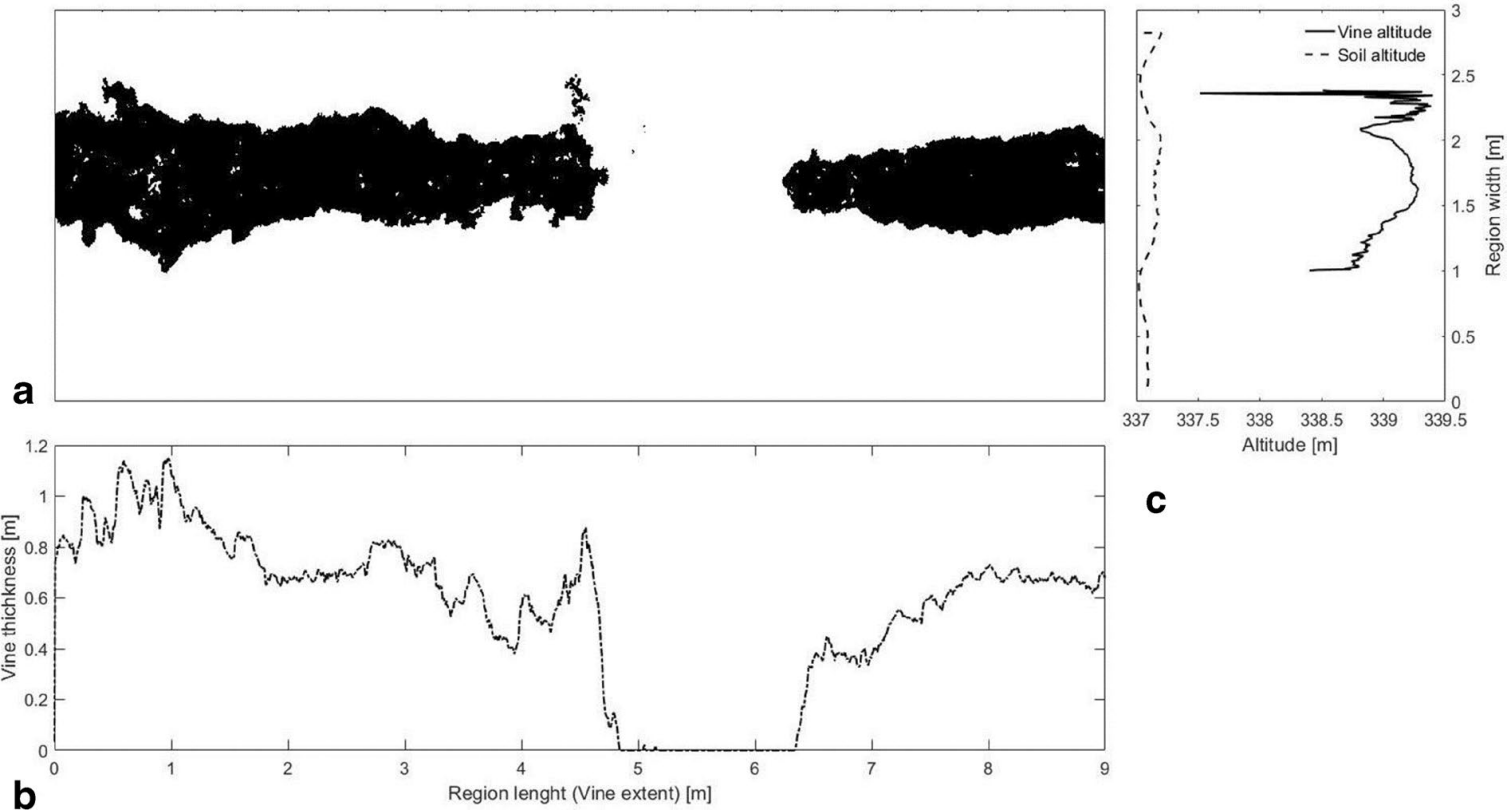
Digital elevation model of vines within a polygon grid and its geometric features. Vine length (**b**) and thickness (**b**) are extrapolated through the binarized image (**a**), mediating the values on the image columns. The soil and vine elevation are obtained mediating respective pixels on horizontal rows (**c**)

Starting from the DEM originated by Agisoft, a uniform polygon grid was then generated to isolate three plants in each vine-row. Case Basse has 3.0 × 1.0 m vine spacing, so each polygon grid was generated starting from the middle point between two rows with regular spacing of 3.0 × 3.0 m, while in Fontone, with a 2.5 × 0.9 m vine spacing, each polygon grid was 2.5 x 2.7 m. Vines and soil were also separated with a thresholding approach. The “*imopath*” function of MATLAB [18] b (Mathworks, Natick, MA, USA) was applied to the DEM to mitigate the effect of terrain slope. Otsu’s thresholding technique was then used to distinguish between soil and vines, generating a logical mask of the complete field [6]. Matlab “*graythresh*” function was used to computes a global threshold from grayscale image, using Otsu’s method. This method chooses a threshold that minimizes the intraclass variance of the thresholded black and white pixels. The global threshold was then used with “*imbinarize*” to convert a grayscale image to a binary image. At this point, the geometric features of vines (vine height $dh$ and mean thickness $\delta_{m}$) in each polygon grid were extrapolated. The DEM of a representative region is shown in Fig. 8a. White pixels identify soil and black pixels identify vine rows.

The sum of black pixels (logical indexing equal to 0) was computed for each column, defining the vine thickness function $$\delta \left( x \right)$$ (in pixels) along the region length. The region length corresponded to the vine extent along the row. This sum was then multiplied for the real spacing associated with each pixel $$dp$$ and the cosine of the angle $$\theta$$ indicating vine slope with respect to the horizontal axis of the original image. The function $$\delta_{p} \left( x \right) = \delta \left( x \right)*dp*{ \cos }\left( \theta \right)$$ represents the distribution of vine thickness with respect to the central axis of the vine row (Fig. 8b). Consequently, the zones where $$\delta_{p} \left( x \right) = 0$$ identifies missing plants. Computationally speaking, missing plants are identified considering all points with $$\delta_{p} \left( x \right) < 0.1$$ m. The remaining set of points was considered as vine thickness. The mean thickness was computed as the mean value of $$\delta_{p} \left( x \right)$$ avoiding missing plants $$\delta_{m} = \left\langle {\delta_{p} \left( x \right)} \right\rangle$$ considering only values with $$\delta_{p} \left( x \right) \ge 0.1$$. With the same approach, the mean elevation of vines and soil was computed for each row of the image. Figure 8c shows the vertical distribution of vine and soil elevation. The mean vine-elevation $$h_{m}^{\left( v \right)}$$ was considered as the first quartile value of $$h^{\left( v \right)} \left( y \right)$$. The mean soil-height $$h_{m}^{\left( s \right)}$$ was extracted as the third quartile value of $$h^{\left( s \right)} \left( y \right)$$, obtaining vine height as $$dh = h_{m}^{\left( v \right)} - h_{m}^{\left( s \right)}$$. 0.8 m was then subtracted from $$dh$$ considering the height of the cordon measured on validation vines. At this point, it was possible to estimate the biomass associated with the complete region $$V_{DEM}$$ and a mean plant $$V_{p}$$. The volume associated with the mean plant was: $$V_{p} = dh*dL*\delta_{m}$$ where $$dL = 0.9$$ is the vine length defined by vine spacing in the vineyard for Fontone and 1.0 m for Case Basse. The volume associated with the DEM was $$V_{DEM} = dh*dA$$, where $$dA$$ is the area of the vines computed by multiplying the total number of black pixel $$N_{px}$$ to the area associated with each pixel, i.e., $$dA = N_{px} *\left( {dp*\cos \left( \theta \right)} \right)^{2}$$. The number of missing plants in the region was identified as: $$N_{ext} = \frac{{L_{v} }}{{\ell_{p} }}$$ where $$\ell_{p}$$ is the length of a plant in the vineyard, equal to $$0.9$$ or 1.0 m according to vine spacing and extension $$L_{v}$$ of the line with missing plant thickness $$\delta_{p} \left( x \right) < 0.1$$ m.

### *3D approach* —*alphashape method*

**Fig. 9:**
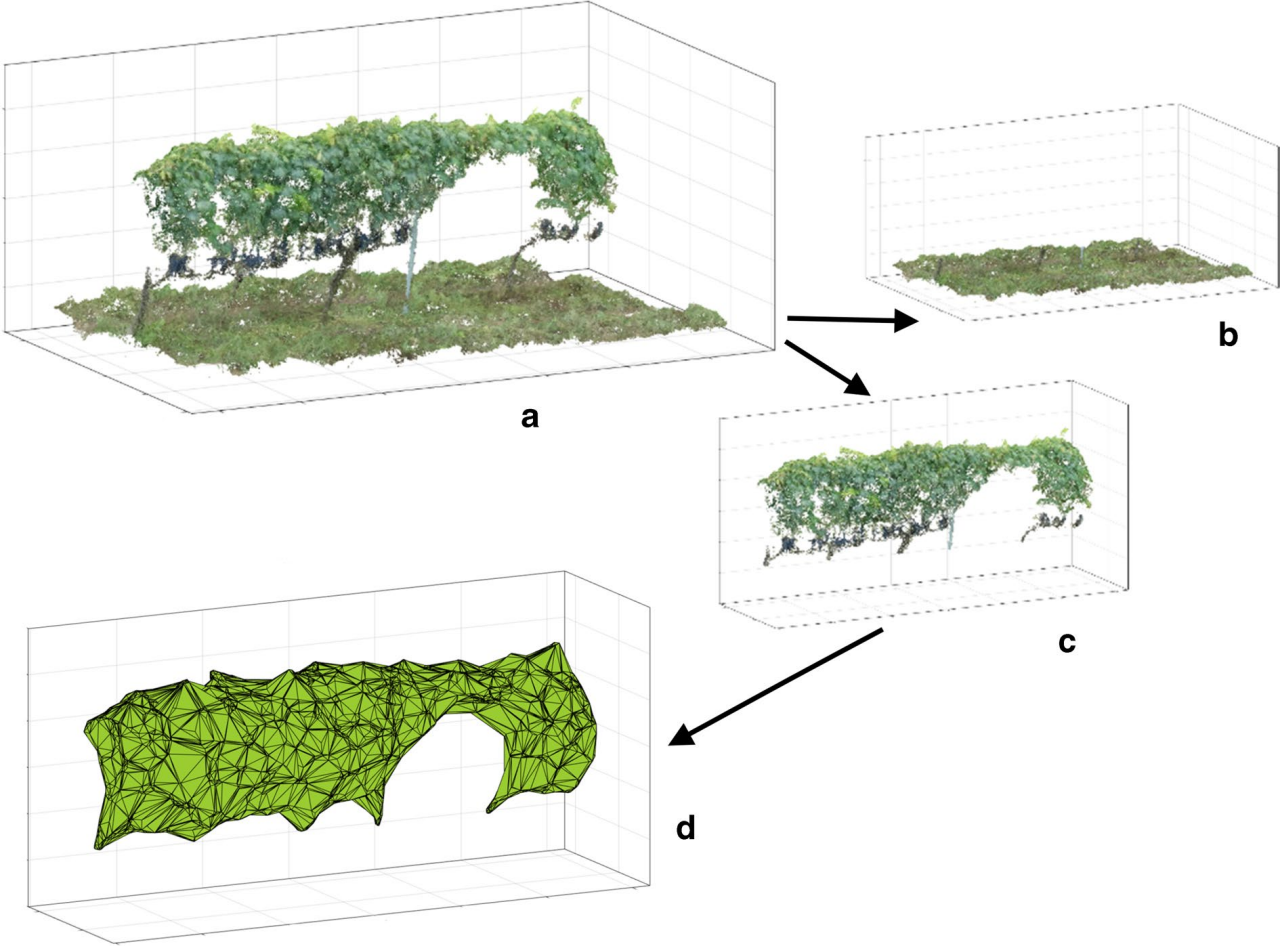
3D dense cloud and alphashape processing of one polygon grid: dense cloud polygon extraction (**a**), ground (**b**) and canopy (**c**) portions, alphashape canopy reconstruction (**d**)

Starting from the 3D dense cloud generated by Agisoft, an alphashape Edelsbrunner and Mucke [11] or volume that envelopes the set of 3D points must be obtained to estimate volumes. The generated dense point clouds were loaded with its original point density to Matlab. Alphashape allows the reconstruction of an object’s shape, namely alphashape, from a set of unorganized points. The parameter α is used to tune the “tightness” of the shape around the points. For a very large value of α, the shape is equivalent to a convex hull. For a very small value of α, the alphashape forms holes and pockets with the shape clustering around the original points. Matlab “*alphashape*” function was used to create a bounding volume that envelops a set of 3-D points. It is then possible to manipulate the alphashape object to tighten or loosen the fit around the points to create a non-convex region and perform geometric queries (Ribeiro et al. [25]). Firstly, the canopy points of the 3D dense cloud were extracted for each polygon grid using a “*planeModel*” object to construct a parametric plane model (Fig. 9a, b, c). Soil and vines were separated using Matlab function “*pcfitplane*” with the parameters equal to 0.5 and 5 for maxDistance and maxAngularDistance, respectively.

The “*alphashape*” function was then applied for each polygon using an α value of 0.5 (Fig. 9d). The function allowed both calculation of the alphashapes and volume estimation. Finally, a volume map was obtained as the union of the volume maps of all sampled rows. To obtain the volume map of a row, the volume value of each section was projected onto the segment joining the starting and ending co-ordinates of the row. For each polygon grid, missing plants are calculated as the total volume within each polygon divided by the average vine volume in the field. For the latter value the average volume of polygon grids where no missing plants were found was divided by three (number of plants in each polygon grid). The resulting average vine volume was 0.39 and 0.31 for Case Basse and Fontone, respectively.

## Data Availability

The datasets used and/or analysed during the current study are available from the corresponding author on reasonable request.
